# Salvianolic acid B inhibits autophagy and activation of hepatic stellate cells induced by TGF-β1 by downregulating the MAPK pathway

**DOI:** 10.3389/fphar.2022.938856

**Published:** 2022-08-04

**Authors:** Na Jiang, Jing Zhang, Jian Ping, Lieming Xu

**Affiliations:** ^1^ Shuguang Hospital Affiliated to Shanghai University of Traditional Chinese Medicine, Shanghai, China; ^2^ Key Laboratory of Liver and Kidney Diseases, Ministry of Education, Shanghai, China; ^3^ Institute of Liver Diseases, Shanghai University of TCM, Shanghai, China; ^4^ Shanghai Key Laboratory of Traditional Chinese Medicine, Shanghai, China

**Keywords:** salvianolic acid B, hepatic stellate cells, autophagy, TGF-β1, MAPK signaling pathway

## Abstract

In liver fibrosis, transforming growth factor-β1 (TGF-β1) can stimulate autophagy and activation of hepatic stellate cells (HSCs). Autophagy, playing a crucial role in HSCs activation, is related to liver fibrosis. Increasing evidence have suggested that antifibrosis effects of salvianolic acid B (Sal B) and their mechanisms of action, however, remain unclear. The aim of the article is to understand the role of Sal B in HSCs autophagy in liver fibrosis. Herein, we demonstrated that inducing TGF-β1 led to dramatic increase in autophagosome formation and autophagic flux in JS1 and LX2, which was mediated through the ERK, JNK, and p38 MAPK cascades. TGF-β1 significantly increased the protein of autophagy and liver fibrosis, including LC3BⅡ, ATG5, α-SMA, and Col.I; Sal B inhibits JS1 autophagy and activation by inhibiting the formation of autophagosomes and autophagic flux. Sal B significantly decreased the LC3BⅡ, ATG5, α-SMA, and Col.I protein expressions; pretreatment with autophagy inhibitors, chloroquine (CQ) and 3-methyladenine (3-MA) or silencing ATG7 further increase these reductions. However, pretreatment with autophagy agonist, rapamycin (Rapa), or overexpressed ATG5 attenuated this decrease. To further assess the importance of this mechanism, the antibody chip was used to detect the change of phosphorylation protein expression of the MAPK signaling pathway after treating JS1 with Sal B. Eleven differentially expressed proteins were verified. Sal B inhibits activation and autophagy of JS1 induced by TGF-β1 through downregulating the ERK, p38, and JNK signaling pathways, as demonstrated by downregulating p-ERK, p-JNK, and p-p38 MAPK protein expressions. In conclusion, Sal B inhibits autophagy and activation induced by TGF-β1 of HSCs possibly by downregulating the MAPK pathway.

## Introduction

The pathological basis of cirrhosis is hepatic fibrosis. The paradigm of HSCs activation remains the foundation for defining event in hepatic fibrosis (Friedman, 2010). Mechanisms of fibrosis have focused on HSCs, which become fibrogenic myofibroblasts during injury through ‘activation’ (Lee et al., 2015), secreting extracellular matrix protein (ECM) (Friedman, 2008) (Higashi et al., 2017). Hence, inactivation of HSCs can lead to enhancement of fibrolytic activity and could be a potential target of antifibrotic therapy (Jung and Yim, 2017). Yet, the continued discovery of novel pathways and mediators, including endoplasmic reticulum stress, oxidative stress, retinol and cholesterol metabolism, epigenetics, receptor-mediated signals, extracellular vesicle (Shuhei Nakamura, 2018), and autophagy, reveals the complexity of HSCs activation (Tsuchida and Friedman, 2017).

Autophagy is an evolutionally conserved cytoplasmic degradation system, in which varieties of materials are sequestered by a double-membrane structure, autophagosome, and delivered to the lysosomes for the degradation (Gao et al., 2020). Due to the wide varieties of targets, autophagic activity is essential for cellular homeostasis (Gao et al., 2020). Autophagy also plays an important role in energy and nutritional metabolism of the liver (Mizushima et al., 2008). The role of autophagy in liver disease depends on the cell type and the stage of the disease (Allaire et al., 2019). The crucial role of autophagy in protecting hepatocyte from death in response to stress induced liver injury, such as acetaminophen (APAP) overdose (Ni et al., 2012) (Li et al., 2019) (Yan et al., 2018) (Shan et al., 2019), liver injury mediated by death receptors (Amir et al., 2013) (Zhong et al., 2016), Wilson’s disease (Polishchuk et al., 2019), and targeting autophagy as a therapeutic strategy for α1-AT deficiency (Mukherjee et al., 2018). Autophagy not only regulates hepatocyte functions but also impacts on non-parenchymal cells such as endothelial cells, macrophages, and hepatic stellate cells (Allaire et al., 2019). A large number of studies demonstrate that the role of autophagy on HSCs activation is complex. Autophagy displays opposite functions depending on the cell type (Allaire et al., 2019). Many studies have shown that selective reduction of autophagy levels of fibrosis-related cells and tissues may be a target for the treatment of liver fibrosis. Activation of HSCs depends on autophagy because the autophagy-mediated degradation of lipid droplets stored in these cells provides energy supply and promotes fibrogenic cell functions (Weiskirchen and Tacke, 2019). Recently, one study showed TGF-β1 treatment increasing both autophagosomes and autolysosomes in LX2 cells, thereby elevating autophagic flux. Ursodeoxycholic acid alleviates experimental liver fibrosis involving inhibition of autophagy flux (Ye et al., 2020). At the same time, many studies have obtained the opposite conclusion. For instance, PDGF induced hepatic stellate cell autophagy inhibiting extracellular vesicle release to attenuate liver fibrosis (Shuhei Nakamura, 2018). Interestingly, we previously showed that TGF-β1 promoted autophagy when it activated HSCs by regulating the MAPK pathway. Sal B has effects on antifibrotic (Liu et al., 2016) and antihepatic injuries (Lin et al., 2015) (Zhang et al., 2017). Our previous research studies have found that Sal B inhibits the ERK pathway *via* inhibiting phosphorylation of MEK and inhibits the p38 MAPK pathway *via* blocking phosphorylation of MKK3/6 and inhibiting expression of MEF2 in HSCs with or without TGF-β1 stimulation. But the role of Sal B on autophagy is unknown.

In the present study, we demonstrate that TGF-β1 can induce HSCs activation and relate to upregulating autophagy (Zhang et al., 2020a). Sal B can inhibit HSCs activation induced by TGF-β1 through increasing autophagic flux by downregulating the MAPK signaling pathway. Meanwhile, this mechanism participates in liver fibrosis progression.

## Material and methods

### Culture of hepatic stellate cells

JS1, the mouse immortalized stellate cell lines, and LX2, human hepatic stellate cell lines were cultured with Dulbecco’s modified Eagle medium (DMEM, GIBCO, United States) supplemented with 10% FBS, penicillin G (100U/mL,GIBCO, United States) and streptomycin (100 μg/ml,GIBCO, United States) with 5% CO_2_ at 37°C. TGF-β1 (recombination human TGF-β1, PeproTech, United States) was added to the media for different times, according to the design of different experiments.

Western blotting. Protein quantification was detected by Western blotting. The antibodies used were as follows: the rabbit antibodies were: Col. I (Abcam,#ab34710), α-SMA (Abcam, #ab32575), Atg5(CST,#12994), Atg7 (CST,#8558), Beclin1 (CST,#3495), LC3B (sigma,#L7543), JNK (CST,#9258), p-JNK (CST,#4671), ERK (CST,#4695), p-ERK (CST,#4370), p38 (CST,#8690), and p-p38 (CST,#4511). The secondary antibodies were horseradish peroxidase (HRP)-conjugated goat antirabbit (LI-COR, #926–68071) or donkey antimouse (LI-COR,#926-32212). ImageJ software was used for quantification. Data were expressed as relative quantification normalized to GAPDH expression and presented as fold change from unstimulated cells.

Reverse transcription-quantitative (RT-q) PCR: RT-qPCR was performed as previously described to determin the gene expression levels in the culture cells. The following primer pairs were used for the qPCR (Table 1).

**TABLE T1:** Primers used for qPCR analysis

Target	Forward primer	Revers primer
Mouse α-SMA	GGAGAAAATGACCCAGATTA	GAGGCGGATGTTCTCAATCT
Mouse Col.Ⅰ	CCAGTGGCGGTTATGACTTC	GCTGCGGATGTTCTCAATCT
Mouse LC3B	AGCAATGGCTGTGTAAGACT	CGCTGGTAACATCCCTTTTT
Mouse ATG5	CCCCAGCCAACAGATTGA	GCCTCCACTGAACTTGACTG
Mouse ATG7	TACGAGCGAGAAGGATTCAC	CTTGATGGAGCAGGGTAAGA
Mouse BECN1	GAGGGATGGAAGGG	GGGCTGTGGTAAGTA
Mouse GAPDH	AAATGGTGAAGGTCGGTGTG	AGGTCAATGAAGGGGTCGTT

### pGMLV-CMV-RFP-GFP-hLC3-Puro lentivirus (purchased from Genomeditech Co., Ltd.) infection of target cells.

The HSCs were seeded in 24-well plates (4 × 10^3^ cell per well) to carry out the pre-infection experiment of the target cells. Before the experiment, different infection holes were set up, according to different MOI, and the amount of lentivirus needed was calculated according to the MOI value and the number of cells. According to the MOI (MOI = 100) value measured by the pre-test, the virus was diluted to the required concentration by using a fresh and complete medium containing 5 g/ml polybrene. After 48 h of infection, each group were incubated with different drugs for corresponding time and then replaced with normal complete medium. Fluorescence changes were observed under a fluorescence inversion microscope and photographed.

Lentiviral autophagy-related gene7(Atg7) short hairpin RNA(shAtg7), which was kindly provided by Professor Mark J. Czaja (Albert Einstein College of Medicine, United States), was cloned into lentiviral vectors. Knockdown and autophagy inhibition was optimal at 5 days after transduction, which was the time point used for the experiments.

### M_atg5 overexpression stable transgenic Strain (purchased from Genomeditech Co., Ltd.)

The primer sequence design was carried out first, and the primers were synthesized according to the designed sequence, then the target gene fragments were amplified and connected to the over-expression vectors after digestion through different restriction sites at both ends. The connecting products were transferred to the prepared bacterial competent cells, and the monoclonal colonies were sequenced, and the correct clones were compared. That is to say, the target gene was successfully constructed and expressed overexpression vector (PGMLV-6395). 293T tool cells were transfected with the transfected overexpression vectors. Western blot was used to verify the effect of overexpression. 293T cells were transfected with the constructed lentivirus vector and packaging mix. The viral solution was collected, concentrated by ultrafiltration, and then the cell titer was determined. JS1 was infected by lentivirus negative control and packaged ATG5 lentivirus. Overexpression of the target gene was detected by qPCR and Western blot (this stable strain is provided by Genomeditech Co., Ltd.).

### Statistical analysis

Results are representative of at least three independent experiments. All data are expressed as the mean ± SD. Statistical analysis was performed using two-tailed Student’s *t*-test between two groups or one-factor ANOVA with Student-Newman-Keuls among multiple groups using SPSS21.0 software. *p* value ≤ 0.05 was considered statistically significant.

## Results

### Sal B inhibits autophagy of HSCs induced by transforming growth factor-β1

**FIGURE 1:**
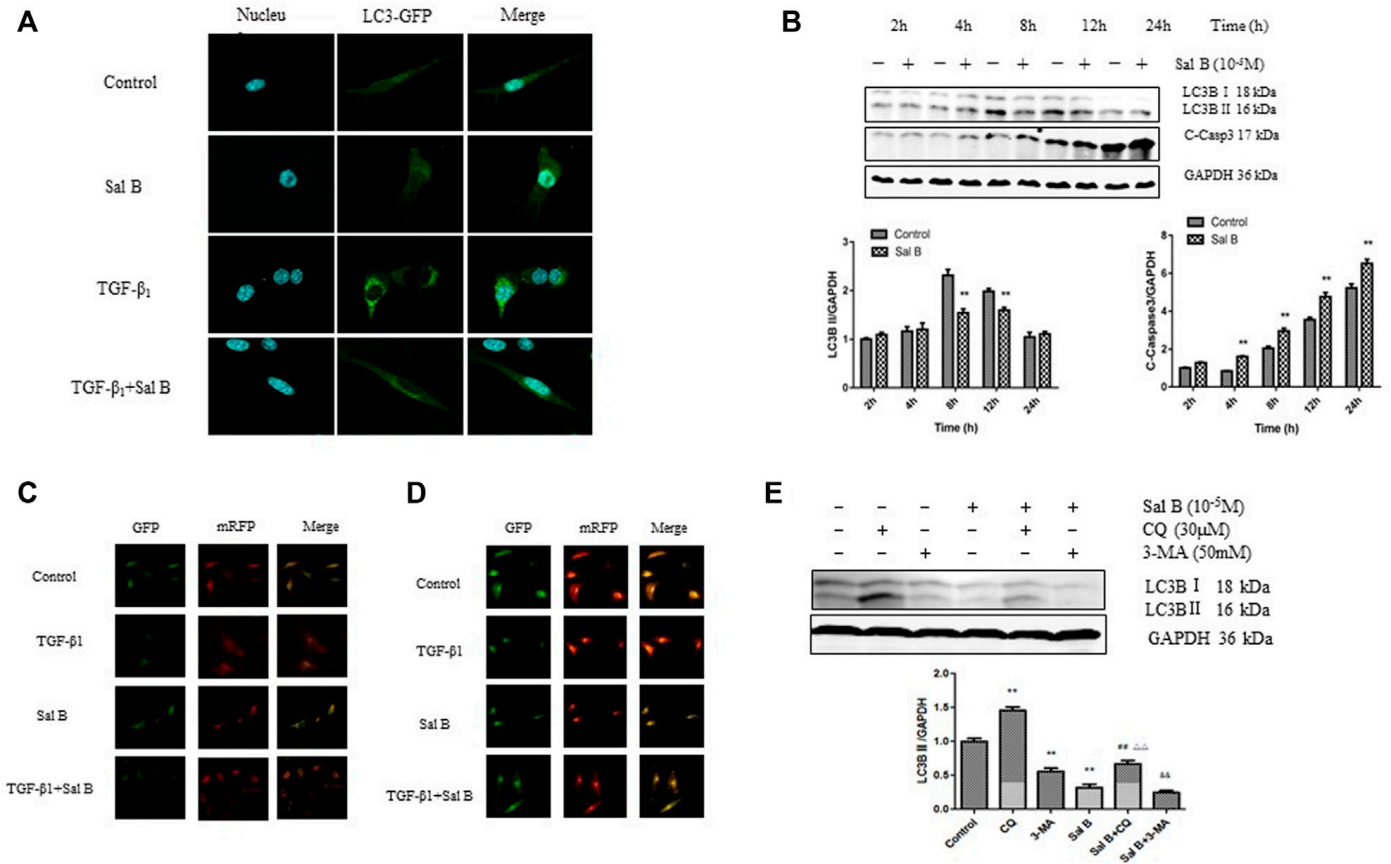
Sal B inhibits the formation of autophagosomes and autophagic flux induced by TGF-β1 in JS1 and LX2**(A)** LC3-GFP plot of JS1 in different groups**. (B)** Expressions of LC3B and cleaved-Caspase 3 in JS1 were detected by WB. **(C,D)** Representative fluorescence inversion microscope images showing GFP (green) and RFP (red) in JS1**(C)** and LX2**(D)**. **(E)** Expression of LC3B in JS1 was detected by WB (*n* = 3 vs*.* control:^∗^ *p* < 0.05,^∗∗^ *p* < 0.01; vs*.* Sal B, ^##^: *p <* 0.01; vs*.* CQ, ^△△^: *p <* 0.01; vs*.* 3-MA, ^&&^: *p <* 0.01) Sal B: salvianolic acid B; C-Casp3: cleaved-caspase3; TGF-β1: transforming growth factor-β1; CQ:chloroquine; 3-MA: 3- methyladenine.

To determine the relationship between Sal B and autophagy activity in liver fibrosis, we detected the expression levels of autophagy-associated protein and autophagic flux in JS1 and (or) LX2. As shown in Figure 1A, JS1 were transfected with an exogenous GFP-LC3B plasmid and then treated with Sal B with or without TGF-β1. The basic autophagy level was lower, LC3-GFP was diffused in the cytoplasm, and only a small amount of green fluorescent dots were observed. GFP-LC3 puncta, which indicates the formation of autophagic vesicles, significantly decreased in the Sal B group compared with the TGF-β1 group in JS1. JS1 were exposed to Sal B at a concentration of 10^−5^M for 2, 4, 8, 12, and 24 h, and the expressions of the autophagy marker LC3B II and apoptosis marker C-Caspase 3 were assessed. As shown in Figure 1B, Sal B could inhibit the protein expression of LC3B II, especially at 8 and 12 h, but induce the expression of C-Caspase 3 at 4, 8, 12, and 24 h.

To investigate whether Sal B regulates autophagic flux in HSCs activation, pGMLV-CMV-RFP-GFP-hLC3-Puro lentivirus was used. We also confirmed that Sal B inhibited autophagic flux by transfecting JS1 and LX2 with the pGMLV-CMV-RFP-GFP-hLC3-Puro lentivirus. The GFP signal is sensitive to the acidic and/or proteolytic condition of the lysosome lumen, whereas RFP is more stable. Therefore, colocalization of both GFP and RFP fluorescence indicates a compartment that has not fused with a lysosome, such as autophagosome. In contrast, an RFP signal without GFP corresponds to an autolysosome (Klionsky et al., 2021). Therefore, the cleaved GFP level can be used to monitor autophagic flux. As expected, incubation of JS1 with TGF-β1 resulted in a marked increase in free GFP, whereas pretreatment with Sal B attenuated the TGF-β1-induced increase of cleaved GFP, suggesting that Sal B inhibited the formation of autolysosome and blocked autophagic flux. The exciting thing is that we observed the same results in LX2 (Figures 1C,D).

We then co-treated JS1 with Sal B and autophagic inhibitors CQ and 3-MA, which blocked the upstream and downstream steps of autophagic flux. As shown in Figure 1E, the protein expression of LC3B II was significantly decreased by 3-MA or Sal B and increased by co-treatment of Sal B and CQ; its level decreased significantly compared with CQ, indicating that Sal B can inhibit the formation of autophagosomes and reduced the total amount of autophagosomes; co-treatment with Sal B and 3-MA: the expression of LC3B II further decreased compared with 3-MA, and it can be considered that the combination of Sal B with 3-MA further reduced the formation of autophagosomes (Figure 1F). These data suggest that Sal B inhibits JS1 autophagy by inhibiting the formation of autophagosomes and autophagic flux.

### Sal B inhibits activation of JS1 through repressing autophagy of JS1 induced by TGF-β1

**FIGURE 2:**
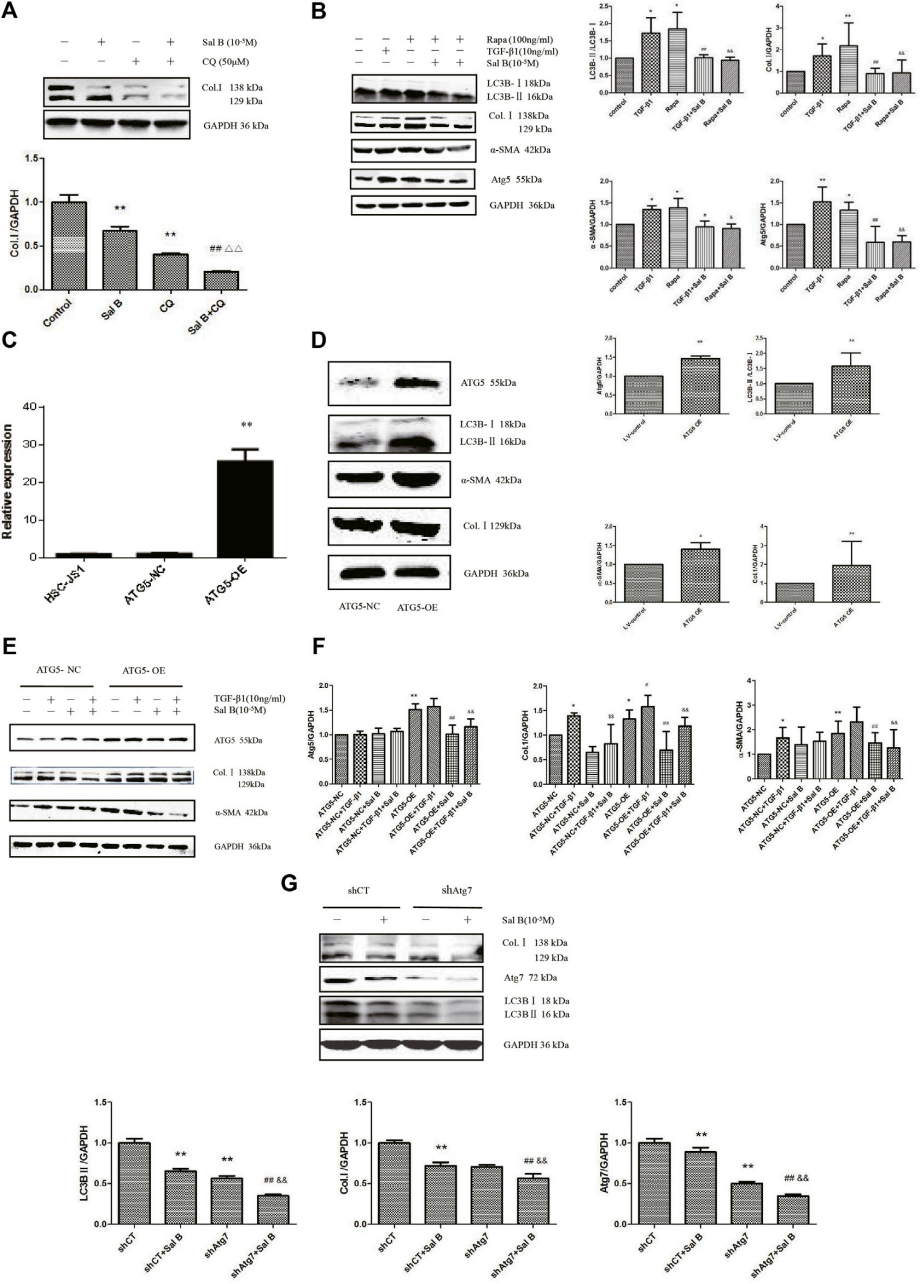
Sal B inhibits JS1 activation induced by TGF-β1 through autophagy. **(A)**Effect of Sal B and CQ, an autophagy inhibitors, on the expression of Col.Ⅰ in JS1.**(B)** Effect of Sal B and Rapa, an autophagy agonist, on the expression of Col.Ⅰ, α-SMA, Atg5, and LC3B.**(C)** Expression of ATG5 mRNA was detected by qPCR. **(D,F)** Effect of ATG5-OE on the expression of Atg5, LC3B, α-SMA, and Col.I. **(E)** Effect of Sal B on the expression of Atg5, Col.I, and α-SMA in overexpression of ATG5 JS1. **(G)** Effect of Sal B on the expression of Col.I, Atg5, and LC3B in interference of ATG7 JS1. *N* = 3 (*N* = 3, vs. control:∗*p* < 0.05,∗∗*p* < 0.01; vs. Sal B, ##: *p* < 0 01; vs. CQ, △△: *p* < 0.01; vs. 3-MA, ^&&^: *p* < 0.01; **(C)** vs. ATG5-NC, ∗∗: *p* < 0.01 **(D,E)** vs. ATG5-NC, *: *p* < 0.05, **: *p* < 0.01 **(F)**vs. ATG5-NC,*:*p* < 0.05,**:*p* < 0.01; vs. ATG5-OE, #:*p* < 0.05, ##: *p* < 0.01; vs. ATG5-NC + TGF-β1, ¥: *p* < 0.05, ¥¥: *p* < 0.01; vs. ATG5-OE + TGF-β1, &: *p* < 0.05, ^&&^: *p* < 0.01. G vs. shCT, **: *p* < 0.01; vs. shAtg7, ##: *p* < 0.01; vs. siCT + Sal B, ^&&^: *p* < 0.01). Col.I: collagen type I; Rapa: rapamycin, α-SMA: α-smooth muscle actin, LC3B: microtubule-associated protein 1 light chain 3B, ATG5: autophagy-related genes; ATG5-OE: overexpression of ATG5; ATG5-NC: empty vector stable cell lines. shCT: control; shAtg7: small hairpin RNA.

In order to investigate whether Sal B inhibit autophagy also contributed to HSCs activation, the following experiments were carried out. As shown in Figures 2A,B, Sal B and CQ markedly suppressed the protein expression of Col.Ⅰ in JS1. Co-treated JS1 with Sal B and CQ further reduced Col.Ⅰ accumulation. At the same time, we found that TGF-β1 and Rapa, a autophagic agonist, could significantly increase LC3BⅡ, Col.Ⅰ, α-SMA, and Atg5 proteins expression, while these proteins were reversed by Sal B, suggesting a possible role of Sal B inhibiting the activation of JS1 by decreasing the formation of autophagosomes.

Preclinical studies have shown that activation of autophagy following selective overexpression of certain ATG protein counteracts disease progression (Allaire et al., 2019). Overexpression of ATG5 extends life in mice and reduces the onset of age-related disease (Jong-Ok Pyo et al., 2013). To further elucidate the role of autophagy on inhibition of HSCs activation by Sal B, ATG5 was overexpressed. We commissioned Genomeditech Co., Ltd. to complete the overexpression of ATG5 (it is called ATG5-OE afterward) and empty vector stable cell lines (ATG5-NC). The expression of mRNA has shown that ATG5 gene expression in wild-type JS1 was similar to that in ATG5-NC, but ATG5-OE was 25.56 times higher than that in ATG5-NC (Figure 2C). It is mean stable transgenic strain of ATG5 gene overexpression is successful. ATG5-OE dramatically upregulated the protein expressions of ATG5, LC3BⅡ, α-SMA, and Col. I (Figure 2D); and next is the treatment of stable transgenic strain with TGF-β1 and Sal B. TGF-β1 could further promote the protein expression of Atg5 and α-SMA compared with ATG5-OE, there was no statistical significance but could significantly promote the protein expression of Col.I. These three proteins markedly decreased by Sal B, indicating an effect of Sal B on autophagy in JS1 activation (Figure 2E). The result of gene expression was similar (Figure 2F).

Then, to further explore the relationship between HSCs activation and autophagy inhibition by Sal B, we established the Atg7 interference model, which was transfected with shAtg7 on the fifth day, and then cells were incubated with 10 ng/ml TGF-β1 or 10^−5^M Sal B. We also demonstrated that Atg7 interference reversed TGF-β1–mediated upregulation of the expression of the Col. I and LC3B II (Zhang et al., 2020a). At the same time, a Western blotting analysis showed that Sal B could further reduce the expression of Col. I, Atg7, and LC3B II (Figure 2G). These results showed that Sal B could further inhibit the autophagy and activation of JS1 under the autophagy defect induced by shAtg7.

Based on the aforementioned experimental results, we draw a conclusion: Sal B inhibits JS1 activation induced by TGF-β1 through autophagy.

### Sal B inhibits autophagy and activation of HSCs by downregulating the ERK, p38, and JNK pathways.

**FIGURE 3:**
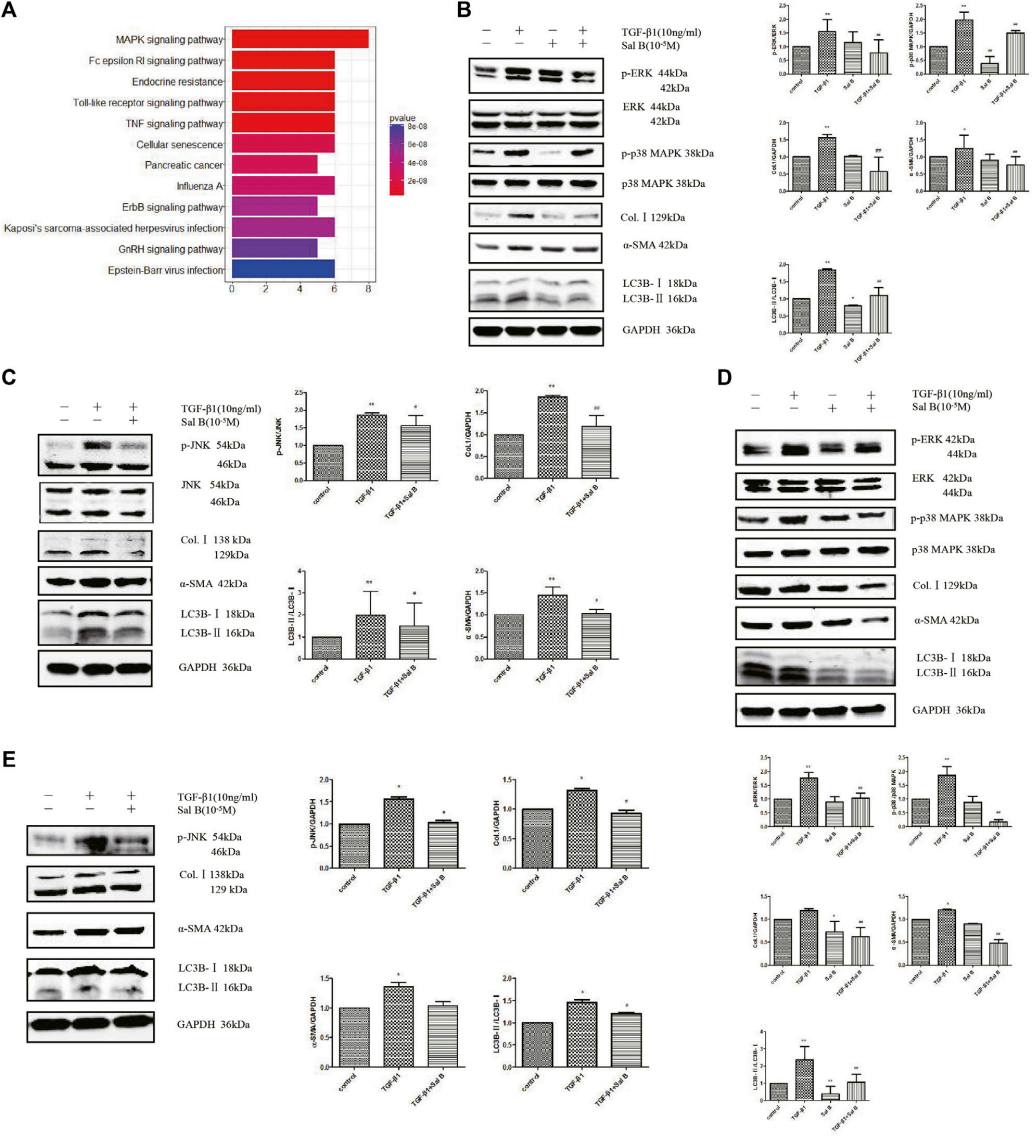
Sal B inhibits activation and autophagy of JS1 and LX-2 induced by TGF-β1 through downregulating the MAPK signaling pathway. **(A)** Protein analysis by MAPK Pathway Phosphorylation Array Kit phosphorylated protein chip (three samples in every group). Expression of p-ERK、p-p38 MAPK、p-JNK、Col.Ⅰ、α-SMA、and LC3B in JS1 **(B,C)** or LX-2 **(D,E)** (vs*.* control:^∗^ *p* < 0.05,^∗∗^ *p* < 0.01; vs*.* TGF-β1: ^#^ *p* < 0.05 ^##^, *p <* 0 01; vs*.* TGF-β1+Sal B:^&^ *p <* 0.05,^&&^ *p <* 0.01).

To explore the mechanism of Sal B inhibiting the autophagy and activation of HSCs, we chose a protein chip from RayBiotech as the research object. The results showed that 11 differentially phosphorylated protein sites expressed were screened out. JNK, p38 MAPK, and its upstream phosphorylated proteins MKK3/6, and upstream phosphorylated proteins of ERK pathways, Mek1, all of them were downregulated (Figure 3A).

Then the aforementioned results are verified. After exposure to TGF-β1 at a dose of 10 ng/ml, the protein expression levels of p-ERK, p-p38 MAPK, p-JNK, Col.Ⅰ, α-SMA, and LC3BⅡ increased compared with controls, whereas these markers were markedly inhibited by Sal B. In addition, ERK, p38 MAPK, and JNK protein expressions remained unchanged **(** Figures 3B,C). Fortunately, we also found the similar results in LX2 **(** Figures 3D,E).

**FIGURE 4:**
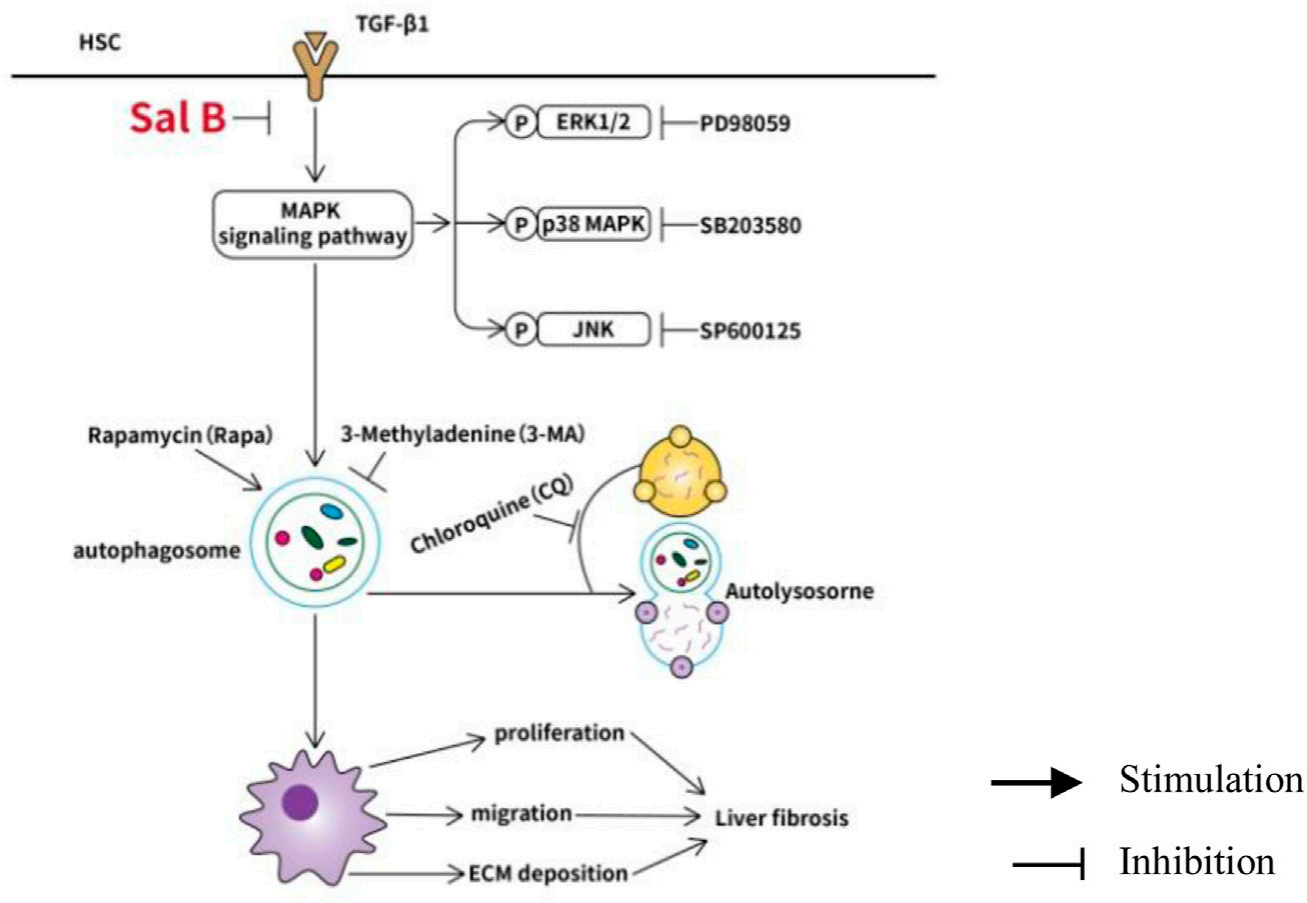
Working model of Sal B inhibits activation and autophagy of HSCs induced by TGF-β1 through downregulating the MAPK pathway.

Collectively, the data indicate that Sal B inhibits activation and autophagy of JS1 in partially mediated ERK, p38, and JNK pathways, and there may be crosstalk between three channels. This possible mechanism of Sal B is shown in Figure 4.

## Discussion

Available evidence suggest Chinese herbal medicine can prevent liver fibrosis. For example, Duhuang Zhechong pill and Gan Shen Fu Fang attenuates liver fibriosis *via* different signaling pathway (Gong et al., 2020) (Du et al., 2020). Among them, Fuzheng Huayu formula has been studied more. Fuzheng Huayu recipe has been used widely for treating liver fibrosis or cirrhosis caused by chronic hepatitis B infection, chronic hepatitis C infection, non-alcoholic steatohepatitis, and so on. A Phase Ⅱ, randomized, multicenter, double-blind, placebo-controlled study was successfully completed to assess the antifibrotic activity of Fuzheng Huayu tablet in patients with chronic hepatitis C plus hepatic fibrosis in the United States in 2013 (Zhang et al., 2020b). But, the mechanisms are unclear. In this research, we revealed a new mechanism of Sal B, a main drug of Fuzheng Huayu formula, inhibiting HSCs activation. We discovered that reducing autophagic flux also play an important role in Sal B inhibiting HSCs activation induced by TGF-β1 through the downregulated MAPK signaling.

TGF-β1 is a potent fibrogenic cytokine that promotes myofibroblast activation and ECM synthesis (Tschumperlin and Lagares, 2020). HSCs activation is stimulated by several cytokines, in particular TGF-β1, mainly released from Kupffer cells, has been supposed to be the cardinal pro-fibrogenic effector of HSCs (Yongfang Gong and Yang, 2020). Many studies have shown that autophagy operates as a critical quality control mechanism for the maintenance of hepatic homeostasis in both parenchymal [hepatocytes (Fang et al., 2021) (Park et al., 2020)] and non-parenchiymal [stellate cells, sinusoidal endothelial cells (Hammoutene et al., 2020), Kupffer cells (Xu et al., 2020)], and cpmpartments (Hazari et al., 2020). We recently showed that TGF-β1 induced autophagy activates hepatic stellate cells *via* the ERK and JNK signaling pathways (Zhang et al., 2020a). Sal B has an obvious effect on antifibrosis. Sal B can inhibit liver fibrosis induced by DMN and CCl_4_ in rats *in vivo* (Tao et al., 2021), and it has a therapeutic effect on liver fibrosis, kidney (Gang Yao et al., 2009), and lung fibrosis (Kesireddy et al., 2019) (Stojanović et al., 2020). Sal B can inhibit the proliferation of lung fibroblasts cell and expression of Col. I, α-SMA, and the production of endogenous TGF-β1 induced by TGF-β1 (Liu et al., 2016). The regulation of Sal B on autophagy varies according to different cell types. For example, Sal B attenuates epithelial–mesenchymal transition in renal fibrosis rats through activating Sirt1-mediated autophagy (He et al., 2020), but in cardiac myocytes, it can inhibit autophagy and protect starving cardiac myocytes (Han et al., 2010). The details presented in our study demonstrate 10^−5^M Sal B could inhibit the activation and proliferation of HSCs induced by TGF-β1. Recent studies suggest that Sal B protects human umbilical vein endothelial cells from oxidative stress at least partially by promoting autophagy *via* activation of the AMPK pathway and downregulation of the mTOR pathway (Gao et al., 2019). Autophagy may interact with NLRP3 activation to contribute to the development of depression, whereas Sal B can promote autophagy and induce the clearance of NLRP3, thereby resulting in neuro-protective and antidepressant actions (Jiang et al., 2017). These results suggest that Sal B may act as an important role in different cells by regulating autophagy. Our study demonstrated Sal B can decrease the expression of LC3BⅡ, a marker of autophagosome, at different times. Meanwhile, we observed cleaved-Caspase 3, the key enzyme to regulate apoptosis, increased at 8, 12, and 24 h. Autophagy appears before apoptosis, which may be related to the inhibition of autophagy to induce apoptosis.

Sal B inhibited autophagy of JS1 induced by TGF-β1 from the perspective. It is suggested that Sal B can reduce autophagosomes induced by TGF-β1. The effect of Sal B on autophagy flux induced by TGF-β1 is studied. In the next experiment, we used pGMLV-CMV-RFP-GFP-hLC3-Puro lentivirus transfected cells to confirm that Sal B inhibited the formation of autolysosome and blocked autophagic flux in JS1. The interesting thing is that we observed the same results in LX2, and then we used 3-MA and CQ to treat JS1, respectively. The results showed that Sal B and autophagy inhibitor could further decrease the expression of LC3BⅡ. Prior studies have demonstrated that the activation of HSCs can be promoted by induction of autophagy. Lipopolysaccharide induced autophagy resulted in LD loss, RA signaling dysfunction to mediate hepatic stellate cell activation (Chen et al., 2017). Recent studies also have suggested autophagy suppression contributes to inhibition of HSCs activation (Yu et al., 2019) (Ma et al., 2019) (Wang L. et al., 2020). Ursodeoxycholic acid alleviates experimental liver fibrosis involving inhibition of autophagy (Ye et al., 2020). This is consistent with our results. However, there are some opposite results (Hu et al., 2020). For example, hepatic stellate cell autophagy inhibits extracellular vesicle released to attenuate liver fibrosis (Shuhei Nakamura, 2018). This may depend on different cell states and different animal models.

In autophagy research, gene of Atg interference (Wang Z. et al., 2020) and overexpression were used (Zhang et al., 2019). Autophagy is highly dependent on some autophagic proteins, among which ATG5 is a very important protein in autophagic body formation. In our experiment, we established the overexpression of ATG5 (ATG5-OE) and empty vector plasmid (ATG5-NC) transfection stable strain. We detected the protein expressions of Col.Ⅰ, α-SMA, Atg5, and LC3BⅡ were significantly increased in ATG5-OE than ATG5-NC. It is suggested that ATG5-OE promotes autophagy and activation in JS1. Then, the study has shown that Sal B can inhibit the activation of HSCs by inhibiting autophagy induced by TGF-β1 and Rapa, as well as ATG5-OE. At the same time, we established the Atg7 interference model. The increase of Col.Ⅰ, Atg7, and LC3BⅡ induced by TGF-β1 was alleviated by shAtg7. Sal B could further reduce the expressions of Col.Ⅰ, Atg7, and LC3BⅡ in the shAtg7 model. Its mean Sal B could further inhibit the autophagy and activation of HSCs under the impaired autophagy induced by shAtg7.

Sal B can inhibit HSCs activation by downregulating autophagy. Next, we will study its mechanism. Our previous research has found (Lv et al., 2010) (Lv and Xu, 2012) (Xu et al., 2012) that Sal B could inhibit HSCs activation induced by TGF-β1 through the MAPK signaling pathway. Qianqian Wang et al. (Wang et al., 2019) reported high glucose caused autophagic cell death by activating the JNK pathway. Previous research had shown autophagy can be regulated by the MAPK signaling pathway (Hanyu et al., 2020) (Chiou et al., 2020). In order to determine the signal pathway of Sal B inhibiting the activation and autophagy of HSCs, we chose a protein chip from RayBiotech. Most focus was on the MAPK signaling pathway. Then, the verification experiments found after exposure to TGF-β1, the level of p-ERK, p-p38 MAPK, p-JNK, Col.Ⅰ, α-SMA, and LC3BⅡ protein increased, whereas Sal B significantly reduced the level of these elevated protein. Fortunately, we also found the similar results in LX2.

In summary, the present study demonstrates Sal B inhibits activation and autophagy of HSCs induced by TGF-β1 partly through downregulating the ERK, p38, and JNK pathways, and there may be crosstalk between these channels. These findings provide a rationale for potential clinical applications of Sal B for the prevention or treatment of liver fibrosis.

## Data Availability

The original contributions presented in the study are included in the article/Supplementary Materials; further inquiries can be directed to the corresponding author.
